# Surveillance and Control of Dengue Fever as a Global Human Treat

**DOI:** 10.34172/jrhs.2024.169

**Published:** 2024-09-30

**Authors:** Abedin Saghafipour, Mohammad Khazaei

**Affiliations:** ^1^Department of Public Health, Faculty of Health, Qom University of Medical Sciences, Qom, Iran; ^2^Department of Environmental Health Engineering, School of Public Health and Research Center for Health Sciences, Hamadan University of Medical Sciences, Hamadan, Iran

## To Editor,

 Dengue fever is considered as one of the most important arboviral diseases and its causative virus includes four serotypes, namely, DEN-1, DEN-2, DEN-3, and DEN-4. It is transmitted to humans by some species of female Aedes mosquitoes.^1^ The symptoms of the disease on a wide spectrum (asymptomatic to symptomatic and fever-free to hemorrhagic) usually begin 5 to 7 days after the bite of an infected mosquito.^2^ The clinical features of the disease are fever with rash, severe headache, eye pain, pain around the eye socket, and muscle and joint pain. This disease is also known as break bone fever because, due to severe pain, the patient imagines that his/her bones are breaking.^1^ Approximately 3.9 billion people in 128 countries are at risk of dengue fever.^3^ In the Eastern Mediterranean region, several outbreaks of dengue fever have been reported in recent years, especially in Pakistan, Saudi Arabia, Yemen, and the United Arab Emirates. Recently, some disease cases have also been reported in the southern and northern provinces of Iran.^4,5^ Aedes aegypti and Aedes albopictus mosquitoes are the main vectors of this disease, and these two species of mosquitoes have been reported in Iran’s neighboring countries, such as Armenia, Turkey, Oman, Afghanistan, and Pakistan^6,7^ and recently reported from Iran.^8^

 Now, the strategies for the surveillance and control of this disease are recommended and classified into two categories, as follows:

## Human surveillance activities

- Teaching prevention tips to people and healthcare personnel: For example, dengue fever is not transmitted from person to person. It is transmitted only through some species of Aedes mosquitoes. Personal protection against mosquito bites is performed using a mosquito net.
- Monitoring human dengue cases is important. Awareness of the warning signs of the disease and referrals for possible diagnosis and treatment should be given to people and healthcare personnel.
- Laboratory-based surveillance: Laboratory-based surveillance and treatment are extremely important.^9^

## Mosquito surveillance activities

 The management of environmental risk factors is important. Aedes mosquitoes usually live in containers and spawn in water collected in abandoned cans, used tires, and the like, and it is better to remove these risk factors from human habitations.

 Employing entomologists in the health departments of universities and collectors forces to collect vector Aedes.

 Entomologists and collectors should be trained in catching mosquitoes and diagnosing invasive Aedes that transmit dengue fever (e.g., Aedes aegypti and Aedes albopictus in Iran).

 Entomological equipment should be prepared; for example, ovitraps for catching mosquito eggs, an entomological dipper for catching mosquito larvae, and a BG-GAT (Gravid Aedes Trap) for capturing adult mosquitoes.^6^

 Entomological checks should be regularly performed to monitor the possible presence of invasive Aedes mosquitoes as potential vectors of disease in the area.

 As a result, it is hoped that with human and entomological surveillance activity, the presence or absence of invasive Aedes mosquitoes will be monitored in all regions of the country. Secondly, with human care and education on methods to prevent the disease, human cases will not be observed, and suspected cases will be quickly found, diagnosed, and treated in time with active case findings.

## Authors’ Contribution

**Conceptualization: **Abedin Saghafipour.

**Investigation: **Abedin Saghafipour, Mohammad Khazaei.

**Supervision: **Abedin Saghafipour.

**Writing–original draft: **Abedin Saghafipour.

**Writing–review & editing: **Abedin Saghafipour, Mohammad Khazaei.

## Competing Interests

 The authors declare that there is no conflict of interests.
